# Alternation of up and down states at a dynamical phase-transition of a neural network with spatiotemporal attractors

**DOI:** 10.3389/fnsys.2014.00088

**Published:** 2014-05-19

**Authors:** Silvia Scarpetta, Antonio de Candia

**Affiliations:** ^1^Dipartimento di Fisica “E. R. Caianiello”, Università di SalernoFisciano (SA), Italy; ^2^INFN Gr. Coll. di SalernoFisciano (SA), Italy; ^3^Dipartimento di Fisica, Università di Napoli Federico IINapoli, Italy; ^4^CNR-SPIN, Sezione di NapoliItaly; ^5^INFN, Sezione di Napoli, Complesso Universitario di Monte S. AngeloNaples, Italy

**Keywords:** criticality, phase transition, STDP, associative memory, spatiotemporal pattern replay, neural avalanches, up and down states

## Abstract

Complex collective activity emerges spontaneously in cortical circuits *in vivo* and *in vitro*, such as alternation of up and down states, precise spatiotemporal patterns replay, and power law scaling of neural avalanches. We focus on such critical features observed in cortical slices. We study spontaneous dynamics emerging in noisy recurrent networks of spiking neurons with sparse structured connectivity. The emerging spontaneous dynamics is studied, in presence of noise, with fixed connections. Note that no short-term synaptic depression is used. Two different regimes of spontaneous activity emerge changing the connection strength or noise intensity: a low activity regime, characterized by a nearly exponential distribution of firing rates with a maximum at rate zero, and a high activity regime, characterized by a nearly Gaussian distribution peaked at a high rate for high activity, with long-lasting replay of stored patterns. Between this two regimes, a transition region is observed, where firing rates show a bimodal distribution, with alternation of up and down states. In this region, one observes neuronal avalanches exhibiting power laws in size and duration, and a waiting time distribution between successive avalanches which shows a non-monotonic behavior. During periods of high activity (up states) consecutive avalanches are correlated, since they are part of a short transient replay initiated by noise focusing, and waiting times show a power law distribution. One can think at this critical dynamics as a reservoire of dynamical patterns for memory functions.

## 1. Introduction

Spontaneous cortical activity, i.e., ongoing activity in the absence of sensory stimulation, can show very complex collective features, with, in some cases, the membrane potential making spontaneous transitions between two different levels called up and down states (Steriade et al., 1993; Cowan and Wilson, 1994; Cossart et al., 2003; Shu et al., 2003). This alternation of “down states” of network quiescence and “up states” of generalized spiking and neuronal depolarization, have been observed to occur spontaneously in a variety of systems and conditions, both *in vitro* (Plenz and Kitai, 1998; Cossart et al., 2003; Shu et al., 2003) and *in vivo* during slow-wave sleep, anaesthesia and quiet waking (Petersen et al., 2003; Luczak et al., 2007) The precise mechanism by which these up states transitions occur is still unclear, but it seems to rely on network mechanisms (Cossart et al., 2003). Up states transitions are almost abolished by pharmacological blockers such as glutamate receptor antagonists (Cossart et al., 2003; Shu et al., 2003) and totally abolished by glutamate and GABA receptor antagonists (Cossart et al., 2003).

Results on *in vitro* and *in vivo* up states has suggested that this spontaneous activity occurred in a highly structured way, with repeating spatiotemporal patterns of cellular activity (Cossart et al., 2003; Luczak and MacLean, 2012). Because of their stereotyped spatio-temporal dynamics, it has been conjectured that network up states are circuit attractors (Cossart et al., 2003). Transitions between down and up states can also be evoked by sensory stimulation (Petersen et al., 2003), and interestingly evoked activity patterns are similar to the up states produced spontaneously (Luczak et al., 2007). Also *in vitro*, in thalamo-cortical slices, the patterns of activity evoked by thalamic stimulation were similar to the patterns of activity that occurred during the up states spontaneously (Luczak and MacLean, 2012).

Many experimental results, both in cell cultures and slices as well as *in vivo* (Gireesh and Plenz, 2008; Petermann et al., 2009; Ribeiro et al., 2010; Plenz, 2012; Haimovici et al., 2013), have also supported the idea that the brain operates near the critical point of a phase transition (Plenz and Thiagarajan, 2007; Chialvo, 2010; Plenz, 2012; Tagliazucchi et al., 2012; Yang et al., 2012; Plenz, 2013; Shew and Plenz, 2013). Neuronal avalanches, i.e., cascade of activity with power law distribution of size and durations (Beggs and Plenz, 2003; Mazzoni et al., 2007; Plenz and Thiagarajan, 2007; Pasquale et al., 2008; Plenz, 2012), are only one of the observed proprieties suggestive of criticality. Criticality is very advantageous for the brain, in terms of optimization of dynamical range, information transmission and capacity (large repertoire of diverse activity patterns) (Kinouchi and Copelli, 2006; Deco et al., 2013; Shew and Plenz, 2013).

All these intriguing results on spontaneous dynamics support the long-lasting hypothesis that brain can move in a landscape with multiple dynamical attractors, and that up states may be the result of the system falling in one of these attractors. From this point of view, the spontaneous fluctuations between up and down state may be the signature of the system posed at a non-equilibrium phase transition, where system fluctuates in the landscape, and flexibly switches from one state to another. Several models have been proposed as explanations for the avalanche power law distributions that emerge in spontaneous cortical activity (Kinouchi and Copelli, 2006; Levina et al., 2007; Plenz and Thiagarajan, 2007; de Arcangelis and Herrmann, 2010; Millman et al., 2010; Lombardi et al., 2012, in preparation; Yang et al., 2012; Scarpetta and de Candia, 2013), and many have discussed the emergence of up and down states in terms of attractor states of a dynamical systems (Holcman and Tsodyks, 2006; Parga and Abbott, 2007; Millman et al., 2010), or self-organized criticality (Lombardi et al., 2012, in preparation). To get bistability, in Parga and Abbott (2007) IF neurons were augmented with a non-linear membrane current, while in Holcman and Tsodyks (2006) and Millman et al. (2010) the crucial role of activity-dependent short-term synaptic depression was pointed out. For example in the attractor model discussed in Holcman and Tsodyks (2006) the mean time the network spends in the down state is comparable to the mean time it takes for the synapses to recover from a certain depressed activity.

In this paper, we study a model that captures not only the emergence of neural avalanches and up and down states, but also additional features of spontaneous activity, such as the stable recurrence of particular spatiotemporal patterns. In particular, recurrence of spatiotemporal patterns has been observed within up states (Luczak and MacLean, 2012, and refs therein), and also neuronal avalanches seem to be highly repeatable, and can be clustered into statistically significant families of activity patterns that satisfy several requirements of a memory substrate (Beggs and Plenz, 2004; Stewart and Plenz, 2006; Gireesh and Plenz, 2008). The model is a network of leaky integrate-and-fire (LIF) neurons, whose connections have synaptic strengths designed in order to store in the network a set of spatiotemporal patterns. The network shows two distinct regimes, a regime of collective replay activity for high connection strength or high noise, and a regime of no activity for low connection strength or low noise. Between these two distinct regimes, it appears a region where noise is able to switch between periods of quiescence (down states) and periods of high rate coherent activity (up states). At a finer temporal scale, within up states, one observes neural avalanches with power law size and duration distributions. In this model, fluctuations between up and down states emerge even in absence of short-term depression, or of any kind of single neuron bistability. It's a network effect, the results of a structured connectivity, that produce multiple dynamical attractors. Near the non-equilibrium phase-transition separating the two regimes in which the network remains permanently in either the up or the down state, one observes high fluctuations, induced by noise, with emergences of transient up states. The mean time the network spends in the down or in the up state is related to noise intensity and connection strength.

## 2. Material and methods

### 2.1. The model

We model the neurons as leaky integrate-and-fire (LIF) units. The postsynaptic membrane potential of neuron *i*, when the neuron does not emit a spike, is given by the equation

(1) $$\frac{dV_{i}(t)}{dt}=-\frac{V_{i}(t)}{\tau_{m}}+\frac{I_{i}(t)}{C},$$

where τ_*m*_ is the characteristic time of the membrane, *C* the membrane capacity, and *I*_*i*_(*t*) the total current input to neuron *i*. The input is given by

(2) $$I_{i}(t)=\sum_{j}\sum_{t_{i}<t_{j}<t}\frac{Q_{ij}}{\tau_{s}}e^{-(t-t_{j})/\tau_{s}}+\sum_{t_{i}<{\hat{t}}_{i}<t}\frac{{\hat{Q}}_{i}}{\tau_{s}}e^{-(t-{\hat{t}}_{i})/\tau_{s}}$$

where *t*_*j*_ are the spike times of neuron *j, Q*_*ij*_ is the total charge released at the synapse between neuron *i* and *j*, τ_*s*_ is the characteristic time of the synapse, ${\hat{t}}_{i}$ are the times of noise events releasing a random charge ${\hat{Q}}_{i}$ at some point of the membrane of neuron *i*, and the sum is extended to the spikes *t*_*j*_ and noise events ${\hat{t}}_{i}$ between the last spike *t*_*i*_ of neuron *i*, and the present time *t*. Defining *J*_*ij*_ = *Q*_*ij*_/[*C*(1 − τ_*s*_/τ_*m*_)] and ${\hat{J}}_{i}={\hat{Q}}_{i}/[C(1-\tau_{s}/\tau_{m})]$, we therefore have (Gerstner et al., 1993; Gerstner and Kistler, 2002)

(3) $$V_{i}(t)=\sum_{j}\sum_{t_{i}<t_{j}<t}J_{ij}\epsilon (t-t_{j})+\sum_{t_{i}<{\hat{t}}_{i}<t}{\hat{J}}_{i}\epsilon (t-{\hat{t}}_{i})$$

where ϵ(*t*) = *e*^−*t*/τ_*m*_^ − *e*^−*t*/τ_*s*_^. When the potential *V*_*i*_(*t*) reaches the threshold value Θ_*i*_, the neuron *i* emits a spike, and its potential is reset to the base value *V*_*i*_ = 0. In the present paper we set the same threshold Θ_*i*_ ≡ Θ for all the neurons, τ_*m*_ = 10 ms, τ_*s*_ = 5 ms, we extract the times ${\hat{t}}_{i}$ of noise events from a Poissonian distribution with a rate ρ = 1 ms^−1^ for each neuron, and extract *Ĵ*_*i*_ from a Gaussian distribution with zero mean and standard deviation $\sqrt{\frac{\alpha }{\rho }\sum_{j}J_{ij}^{2}}$. The constant α, which has the dimension of a rate, sets the “noise level” of the network.

The synapse strengths *J*_*ij*_ are held fixed during the simulation (no short term plasticity). They are set at the beginning with a “learning procedure” (Scarpetta et al., 2001, 2002; Scarpetta and Marinaro, 2005; Scarpetta and Giacco, 2012; Scarpetta et al., 2013), inspired to spike time dependent plasticity (STDP) (Markram et al., 1997, 2011). During this initial “learning procedure,” we store *P* patterns in the network connections. A pattern μ = 1, …, *P* is a phase-coded spike train of period *T*^μ^, with one spike per neuron and per cycle, where the activity of neuron *i* is given by

(4) $$x_{i}^{\mu }(t)=\sum_{n=-\infty }^{\infty }\delta \left[t-(t_{i}^{\mu }+nT^{\mu })\right].$$

**Figure 1:**
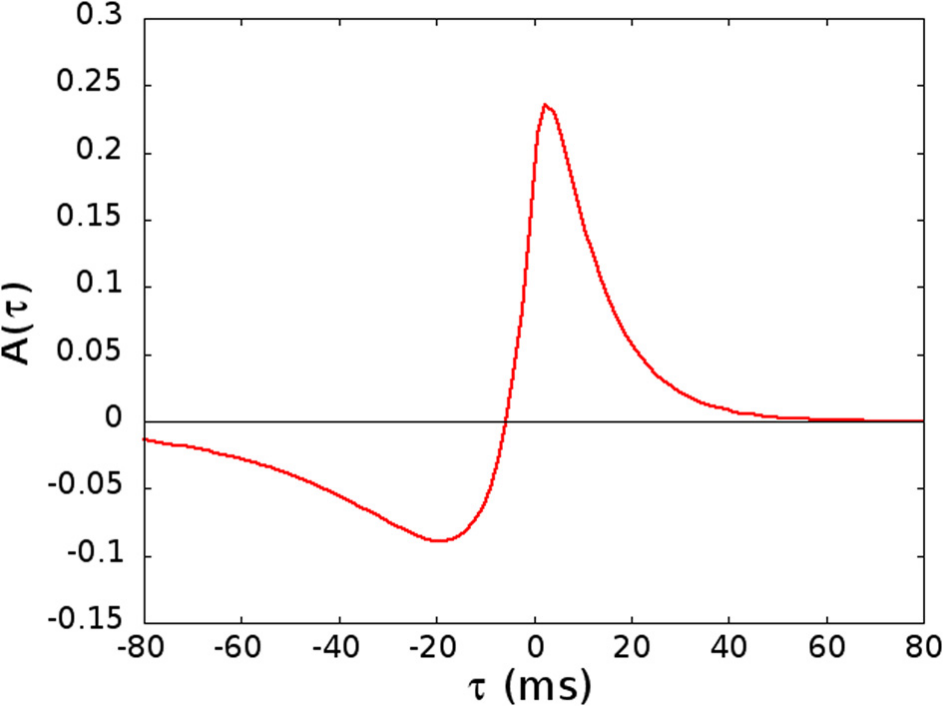
**The kernel *A*(τ) used in Equation (5), given by *A*(τ) = *a*_*p*_*e*^−τ/*T*_*p*_^ − *a*_*D*_*e*^−η τ/*T*_*p*_^ if τ > 0 and *A*(τ) = *a*_*p*_*e*^ητ/*T*_*D*_^ − *a*_*D*_*e*^τ/*T*_*D*_^ if τ < 0, with *a*_*p*_ = [1 + η*T*_*p*_/*T*_*D*_]^−1^, *a*_*D*_ = [η + *T*_*p*_/*T*_*D*_]^−1^, *T*_*p*_ = 10.2 ms, *T*_*D*_ = 28.6 ms, and η = 4**.

The times *t*^μ^_*i*_ are given by $\left(\frac{\phi_{i}^{\mu }}{2\pi }\right)T^{\mu }$, where ϕ^μ^_*i*_ are phases chosen randomly in [0, 2π) and then kept fixed, that give the “order of spiking” of neurons within pattern μ. Therefore, during the initial learning procedure, the network is forced to replay pattern μ, and the connections evolve due to STDP, so that in the interval [−*T*, 0] the change in the connection *J*_*ij*_ is given by

(5) $$\begin{array}{l} \delta J_{ij}=H_{i}\left(\frac{T^{\mu }}{T}\right)\int_{-T}^{0}\text{​}\text{​}dt\int_{-T}^{0}\text{​}\text{​}dt^{\prime }x_{i}(t)A\left(t-t^{\prime }\right)x_{j}\left(t^{\prime }\right)\\ \;=H_{i}\sum_{n=-\infty }^{\infty }A\left(t_{j}^{\mu }-t_{i}^{\mu }+nT^{\mu }\right). \end{array}$$

where *H*_*i*_ is a constant depending on the postsynaptic neuron *i* that sets the strength of the connections, $\frac{T^{\mu }}{T}$ is a normalization factor, *x*_*j*_(*t*) is the activity of the presynaptic neuron at time *t*, and *x*_*i*_(*t*) the activity of the postsynaptic one. In STDP, the learning window *A*(τ) is the measure of the strength of the synaptic change when a time delay τ occurs between pre and post-synaptic spikes. The window *A*(τ) is the one introduced and motivated by Abarbanel et al. (2002), with the same parameters used in Abarbanel et al. (2002) to fit the experimental data of Bi and Poo (1998), see Figure 1. This function satisfies the balance condition ∫^∞^_−∞_ *A*(τ) *d*τ = 0. Notably, when *A*(τ) is used in Equation (5) to learn phase-coded patterns with uniformly distributed phases, then the balance condition assures that the sum of the connections on the single neuron ∑_*j*_ *J*_*ij*_ is of order $1/\sqrt{N}$, and therefore, it assures a balance between excitation and inhibition (Scarpetta et al., 2010). Note that, as we are studying a network of excitatory neurons, the negative connections have to be thought as connections mediated by fast inhibitory interneurons. When multiple phase-coded patterns are stored, the learned connections are simply the sum of the contributions from individual patterns, namely,

(6) $$J_{ij}=\sum_{\mu =1}^{P}\delta J_{ij}^{\mu }.$$

Throughout the paper we use a number of neurons *N* = 3000, a period *T*^μ^ = 333 ms, and a number of patterns *P* = 2. Moreover we use two values for the strengths *H*_*i*_ of the connections in Equation (5). A value *H*_0_ for “normal” neurons, and a value *H*_1_ = 3*H*_0_ for “leader neurons,” that are chosen for each pattern μ as a fraction of 3% of the neurons that have consecutive phases, for example the lowest phases in the interval [0, 2π). Note that the values of *H*_0_ are expressed in units of the threshold Θ of the neurons. The role of these few “leader” neurons, with higher incoming connection strenghts, is that of collect and amplify activity initiated by noise, and give rise to a cue able to initiate the short collective replay.

After the learning procedure, we perform a pruning procedure, by which only a fraction of the *N*(*N* − 1) connections *J*_*ij*_ survives. Namely, for each neuron *i*, we take all the incoming connections *J*_*ij*_, and separately consider the positive (excitatory), and the negative (inhibitory) ones. As for the positive ones, we delete a fixed fraction *f*^+^_prune_ of them that have the lowest value. Then, we delete a fraction *f*^−,*i*^_prune_, that can depend on the neuron *i*, of the negative connections that have the lowest (absolute) value, choosing *f*^−,*i*^_prune_ so that the sum of the incoming connections to neuron *i* at the end is as close as possible to zero. Throughout the paper we use *f*^+^_prune_ = 70%. As a consequence, at the end of the pruning process, about 12% of the *N*(*N* − 1) connections survived as positive connections, and 27% as negative connections, with statistical fluctuations of order of $1/\sqrt{N}$. After the learning and pruning procedure is applied, the dynamics of the network is studied with the connections *J*_*ij*_ fixed, that is we do not apply STDP nor short term depression.

## 3. Results

We studied the dynamics of the network with *N* = 3000 neurons as a function of two parameters, the parameter *H*_0_ setting the strengths of the connections, and the parameter α setting the noise level. The former is expressed in units of the threshold Θ of the neurons, while the latter has dimensions of ms^−1^. We started with a network with all the potentials *V*_*i*_(0) = 0, and let the system evolve subjected to Equation (1). We discarded the first 60 s of the dynamics, to avoid considering the transient, and analyzed the dynamics for a total of 10^7^ spikes, or 1200 s, whichever condition was met before^1^. The simulated time was therefore between 180 and 1200 s, depending on the average spiking rate of the neurons. For each value of the pair of parameters *H*_0_ and α, we average the results over four realizations of the patterns, that is of the quenched random phases ϕ^μ^_*i*_.

### 3.1. Spiking rate distribution and dynamical regimes

**Figure 2:**
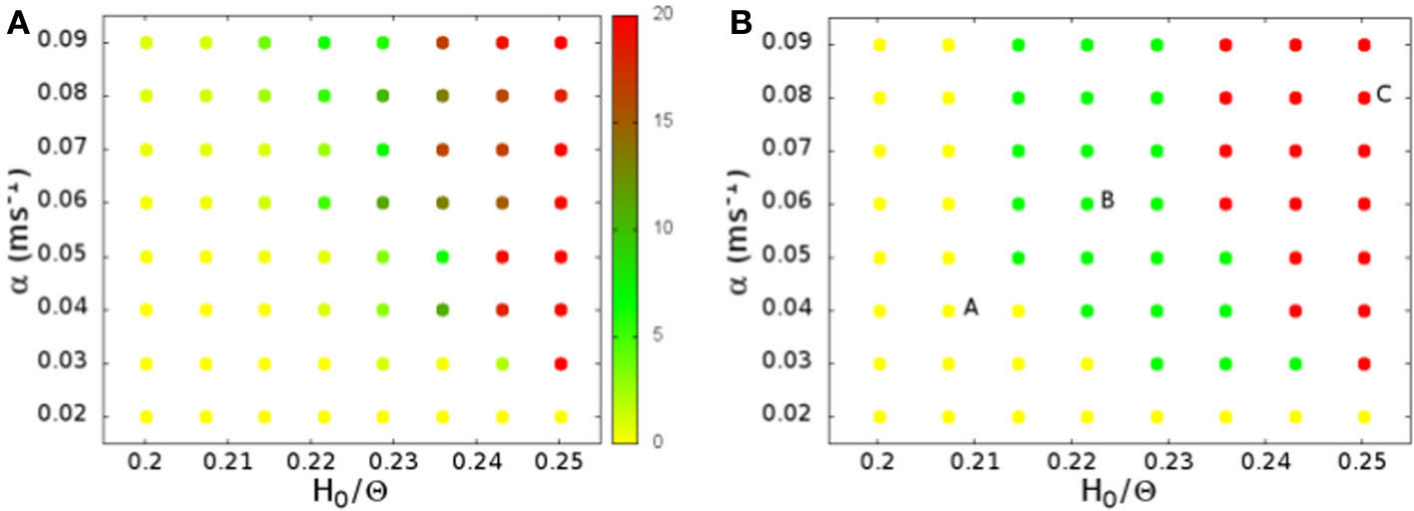
**(A)** The average spiking rate in Hz per neuron, as a function of the noise level α and the connection strengths *H*_0_. **(B)** Shape of the firing rate distribution: nearly exponential (yellow), nearly Gaussian (red), or bimodal (green). The letters on the plot mark the points whose rate distribution is shown in Figure 3, and whose activity is shown as a raster plot in Figure 4. As a function of the noise level α and the connection strengths *H*_0_ we identify a region with bimodal rate distribution with alternation of up and down states (green), which separates the two distinct regimes of low nearly exponential firing rate (yellow) and the regime with nearly Gaussian high firing collective activity (red).

**Figure 3:**
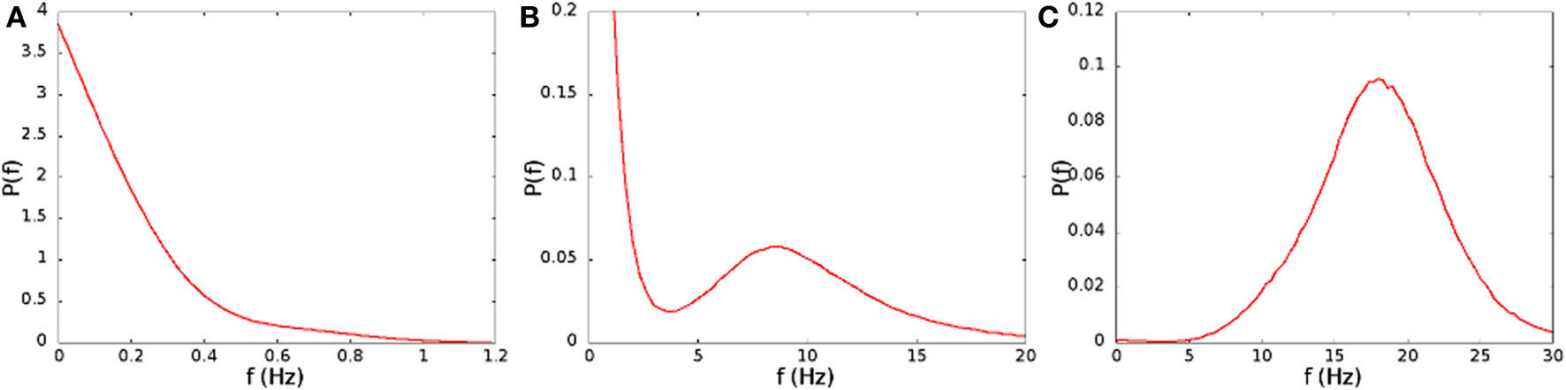
**Distribution of the spiking rates on time intervals of 1 ms, for (A) *H*_0_/Θ = 0.207, α = 0.04 ms^−1^, (B) *H*_0_/Θ = 0.221, α = 0.06 ms^−1^, (C) *H*_0_/Θ = 0.250, α = 0.08 ms^−1^**. These parameters correspond to the points marked with the letters A, B, and C in Figure 2B.

In Figure 2A we show the average spiking rate in Hz per neuron, as a function of the noise level α and the connection strengths *H*_0_. While the average rate increases continuously as either of the parameters is increased, the distribution of the rates in a finite interval of time changes qualitatively. We bin the time in 1 ms intervals, evaluate the rate in Hz per neuron for every interval, and compute the distribution of the rates. For low connection strength, or low noise, the distribution is nearly exponential (see Figure 3A), with an average rate lower than 2 Hz. For high connection strength and high noise, the distribution is nearly Gaussian (see Figure 3C), with an average rate higher than 13 Hz. In an intermediate region the distribution is bimodal (see Figure 3B), and shows both peaks, one exponential at low rates, and one Gaussian at high rates with a minimum in the distribution. This three different regimes are shown in Figure 2B with different colors. The intermediate bimodal regime resembles the phase coexistence observed in a first order equilibrium phase-transition, even though in our case the transition is a non-equilibrium one.

**Figure 4:**
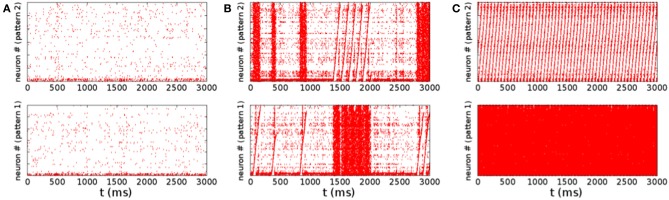
**Raster plots for the same three set of parameters of Figure 3**. The same identical spike train is shown two times, in the up and down panel, corresponding to two different sorting of the neurons on the vertical axis. The sorting order is done according to the order of pattern number 2 in the upper panels, while sorting is done with respect to pattern number 1 in the lower panel. Emergence of collective dynamics which replays one of the pattern appears in the raster plot like a sawtooth, if the neurons are sorted on the vertical axes in the order to the same pattern that is being replayed. Low rate activity is shown in **(A)**, corresponding to the nearly exponential rate distribution shown in Figure 3A. Alternation of states of quiescence with states of higher activity is shown in **(B)**, corresponding to Figure 3B. During the states of higher activity a collective coherent replay of one of the two stored patterns emerges. In this regime the noise is able to initiate a short collective replay of a pattern, and also to stop it. In the picture **(B)** we can see that both patterns are initiated intermittently, a short replay of pattern 2 is followed by a quiescence period and then by a short pattern 1 replay. Raster plot in picture **(C)** shows a regime with stable attractors, with permanent replay of pattern 2.

The qualitative difference in the distribution of the spiking rates, corresponds to a different dynamical behavior. At low rates, when the distribution is nearly exponential with a maximum at zero rate, the dynamical behavior is dominated by noise. The potential of neurons is governed by a Ornstein–Uhlenbeck process, and with some probability crosses the threshold giving rise to a spike, that is not able, however, to generate a spreading activity in the network (Figure 4A). On the other hand, in the high rate regime, the noise triggers the replay of one of the patterns encoded in the network (Figure 4C). In this case, once the replay of the pattern has started, the noise is not able to stop it, so that the replay is permanent^2^. The intermediate regime, corresponding to a bimodal distribution of the rates, is shown in Figure 4B. In this case the noise is able to start the replay of a pattern, but also to stop it, so that the activity is intermittent, and resembles the experimentally observed alternation of up and down states. The firing rate is high in the “up” state (during short replays) and is low during “down” states, therefore the distribution of the rates is bimodal. The corresponding region, shown with green dots in Figure 2B, separates the regimes of permanent collective replay of spatiotemporal pattern (red) and the region of quiescence with low activity (yellow). Such non-equilibrium phase transition has been recently studied in a similar model (Scarpetta and de Candia, 2013, 2014) showing that in the region where the order parameter, which measures the similarity between spontaneous dynamics and the stored dynamic patterns, passes from zero to one, the fluctuations of the order parameter are maximized.

Note that the replay of a pattern appears in the raster plot of Figure 4 as a sawtooth, when the neurons are sorted on the vertical axes in the order of the pattern that is being replayed. On the other hand, it appears as completely random when the neurons are sorted in another way, for example in the order of a pattern that is not being replayed. The alternation of states seen in the raster plot in the bimodal region (Figure 4B) resembles the alternation of up and down states observed to occur spontaneously. Notably, as reviewed in Luczak and MacLean (2012), there are experimental evidences that during the up states often neurons activate in a surprisingly similar sequential order, reproducing default spatiotemporal patterns.

### 3.2. Avalanches size and time distribution

In the intermediate regime, where the network alternates between up and down states, we observe that inside the periods of high firing rate (up states), at a finer level, the activity is made of a series of cascades or “avalanches,” separated by short drops in the rate. Cortical activity cascades that follow precise power laws, i.e., neural avalanches, have been observed experimentally during spontaneous cortical activity *in vitro* and *in vivo* (Plenz, 2012, and references therein).

Experimentally neural avalanches are defined in terms of local field potential recorded at electrodes, that average the activity of many neurons. In our model, we have to distinguish between the few spikes generated by noise, that we want to characterize as no activity, and the spikes generated when a collective pattern is replayed, that represent instead an activity in the network. Due to the separation we have seen on the global spiking rates, with rates lower than 2 Hz corresponding to no activity, and rates larger than 13 Hz representing the collective replay of a pattern, we identify “avalanches” as consecutive time bins with a rate higher than a threshold *R*_min_ = 7 Hz. Successive time bins are concatenated until an empty bin (rate lower then *R*_min_) is reached, at which the concatenation process stops.

**Figure 5:**
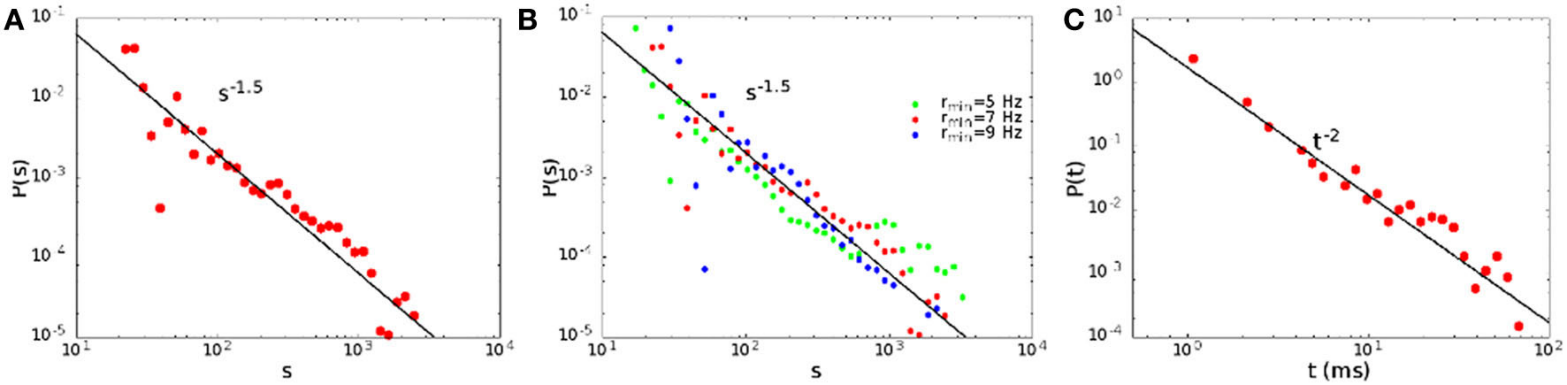
**The size (A) and duration (C) distribution of the avalanches for *H*_0_ = 0.221, α = 0.06, point B in Figure 2B**. In **(B)** we show the dependence of the size distribution on the threshold rate *R*_min_.

We define the size of an avalanche as the total number of spikes, that is the integral of the rates over the avalanche duration. In Figure 5 we show the distribution of the sizes (A) and durations (C) of the avalanches for *H*_0_/Θ = 0.221, α = 0.06, that corresponds to point B in Figure 2B. Note that the distributions are well described by power laws, with exponent 3/2 for the sizes and 2 for the durations, as experimentally observed (Plenz, 2012). Such a behavior is quite robust, and is observed generically in the region of the non-equilibrium transition between the replay and non-replay of spatiotemporal patterns (Scarpetta and de Candia, 2013). It does not depend on the precise value of the *R*_min_ chosen. Indeed, as shown in Figure 5B, the size distribution follows approximately the same power law in a range of *R*_min_ from *R*_min_ = 5 Hz to *R*_min_ = 9 Hz.

### 3.3. Waiting times between avalanches and up and down states

**Figure 6:**
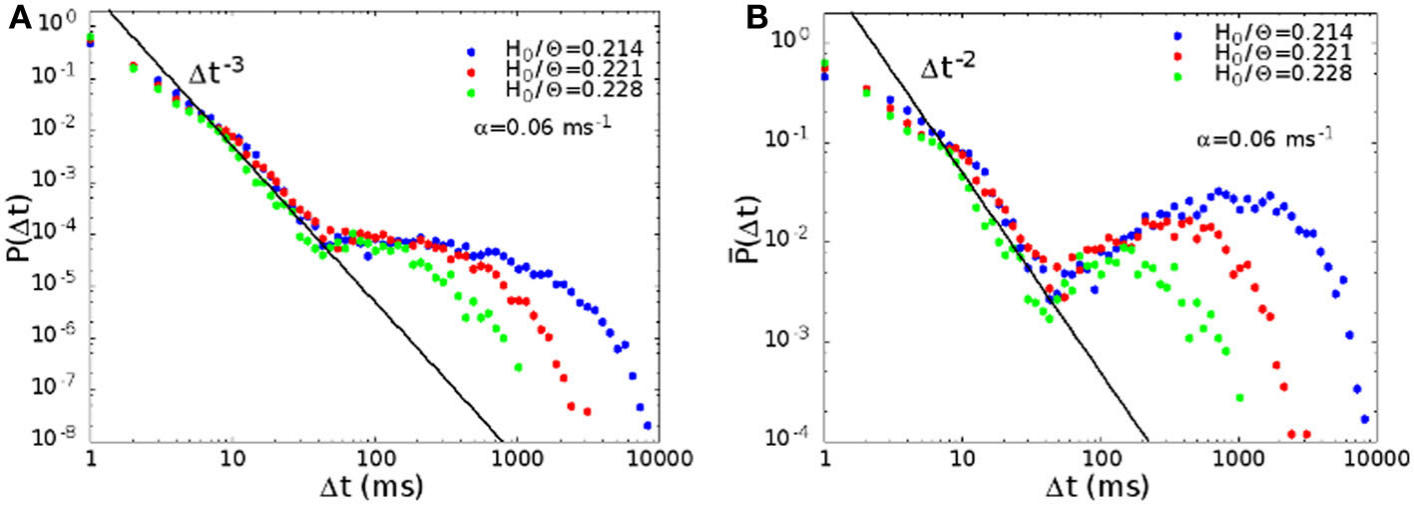
**(A)** Waiting time distribution *P*(Δ*t*) between avalanches, for *H*_0_/Θ = 0.214, 0.221, and 0.228, and α = 0.06 ms^−1^, that is for parameters where the rate distribution is bimodal. The middle curve (red) for *H*_0_/Θ = 0.221 corresponds to point B in Figure 2B, while the green and blue curves to points next to point B to the right and to the left. **(B)** Same data of **(A)**, with a different definition of the probability distribution *P*(Δ*t*) (see text).

We have computed the distribution *P*(Δ*t*) of the waiting times between successive avalanches. In Figure 6A we show the distribution for synaptic strength *H*_0_/Θ = 0.214, 0.221, and 0.228, and noise α = 0.06 ms^−1^, in the region where the rate distribution is bimodal. The middle (red) curve at *H*_0_/Θ = 0.221 corresponds to point B in Figure 2B. The distribution presents a regime between 10 and 50 ms characterized by a power law with exponent −3, preceded by a regime with a lower slope. For times larger than 50 ms, the distribution shows a broad plateau, that is longer the lower the noise, or the strength of the connections.

A power law regime in the waiting times between avalanches has been observed also experimentally, for example in freely behaving rats (Ribeiro et al., 2010), or in cortical slices (Lombardi et al., 2012, in preparation). The second regime, corresponding to large waiting times, is also observed in Lombardi et al. (2012, in preparation).

The power law regime corresponds to waiting times between successive avalanches within the same up state. The power law in the distribution indicates temporal correlation, i.e., that consecutive avalanches belonging to the same up state are correlated. Indeed in our model the up state is the result of the system falling in one of the many metastable spatiotemporal pattern attractors, corresponding to a collective replay activity. The large bump at long times in the waiting time distribution is related on the other hand to down states, that is intervals in which the network does not replay any of the encoded patterns.

We have plotted data of Figure 6A also in an alternative way. While *P*(Δ*t*)δ*t* is the probability of observing a waiting time between Δ*t* and Δ*t* + δ*t*, in Figure 6B we plot *P*(Δ*t*), where *P*(Δ*t*)δλ is the probability of observing a waiting time between Δ*t* and Δ*t*(1 + δλ). Note that *P*(Δ*t*) = *P*(Δ*t*)Δ*t*. With this alternative definition, the distribution becomes non-monotonic, with a pronounced maximum at high values of Δ*t*, and the exponent of the initial power law becomes −2.

**Figure 7:**
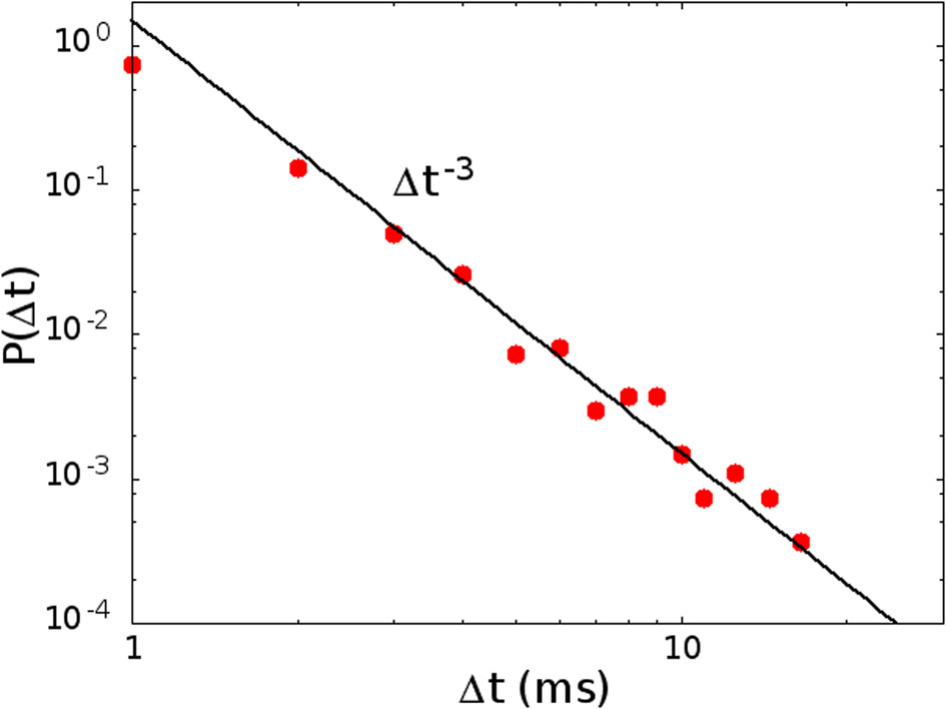
**The distribution of waiting times *P*(Δ*t*) in the region of high activity, for *H*_0_/Θ = 0.250, α = 0.08 ms^−1^ (point C in Figure 2B)**. In this case the plateau at high values of the waiting time, corresponding to down states, disappears, because the replay of the patterns becomes continuous, and only the power law, corresponding to the concatenation of correlated avalanches inside an up state, is observed.

In Figure 7 we show the distribution *P*(Δ*t*) in the region of high activity, marked in red in Figure 2B. In this case the replay of the pattern becomes continuous, and therefore the plateau at long times, in the distribution of waiting times, disappears. The distribution therefore shows only the power law regime, as shown in Figure 7, corresponding to the point in phase space marked with letter C in Figure 2B. On the other hand, in the region of low activity, marked in yellow in Figure 2B, avalanches become very sparse, so that the distribution of waiting times is different from zero only for very long times, and in this case the initial power law regime disappears.

As in Lombardi et al. (2012), we define the up states as periods of high activity characterized by the concatenation of consecutive avalanches with waiting times lower than *T*_max_ = 50 ms, the maximum time falling inside the power law regime of waiting times. Successive avalanches are concatenated until a waiting time larger than *T*_max_ is reached, at which the concatenation process stops. Similarly, down states are defined as a concatenation of waiting times larger than *T*_max_. An isolated avalanche preceded and followed by a waiting time larger than *T*_max_ does not stop the down state.

**Figure 8:**
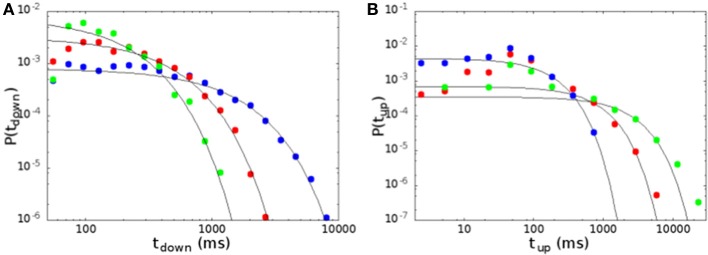
**Distribution of the durations of down (A) and up (B) states, for the same parameters of Figure 6**. Continuous lines are exponential fits to the distributions. Durations of down states are well fitted by an exponential, while durations of up states show some deviations both at short and at large times.

In Figure 8 we show the distribution of the durations of down and up states, for the same parameters of Figure 6. The distribution of durations of down states (Figure 8A) is well fitted by an exponential (continuous lines in the figure), showing that the transition from down to up states is controlled by a Poissonian probability, due to the noise focusing that triggers a replay of one of the patterns encoded in the network. On the other hand, the distribution of durations of up states cannot be fitted by an exponential as well as the one of down states, except for a narrow interval at large times. Indeed, one observes an excess of durations around 100 ms, that corresponds in the model to the duration of one period of the replayed pattern. Moreover, when one approaches the region of parameter space where the replay of the patterns becomes continuous, the distributions show significant deviations from the exponential also for large times, and could be better fitted by a stretched exponential. This is apparent going from blue to red and green curves in Figure 8B, that are all in the bimodal region, but get closer and closer to the region of self-sustained replay. Note that, when one goes deep inside the region of self-sustained replay, no down states are observed in practice, and up states last for a time of the order of the experimental time.

### 3.4. Behavior in presence of short term depression

It has been conjectured that the alternation between up and down states depends crucially on the short term synaptic depression (STSD). As we have shown, in our model this instability between up and down states is present even in absence of short term depression, and is due instead to the particular structure of connections, that are far from being random. Such structure determines in the network a large transition region of phase space, where there is a co-presence of both dynamical attractor states, corresponding to the replay of the patterns encoded, and the attractor corresponding to quiescence of the network.

However, as short term depression is present in real synapses in the brain, we show here that it does not invalidate the behavior displayed by the model considered here, but changes only the parameters, such as the strength of connections, where the transition region appears. We have added STSD in the model, implementing a dynamics on the connections *J*_*ij*_ according to the equation

$$\frac{dJ_{ij}}{dt}=\frac{1}{\tau_{r}}(J_{ij}^{0}-J_{ij}),$$

where *J*^0^_*ij*_ are the connections given by Equation (6), and τ_*r*_ = 10 ms is the recovery time of synapses. Moreover, we depress *J*_*ij*_ by a factor *f*_stsd_ = 0.5, every time the presynaptic neuron *j* fires a spike.

**Figure 9:**
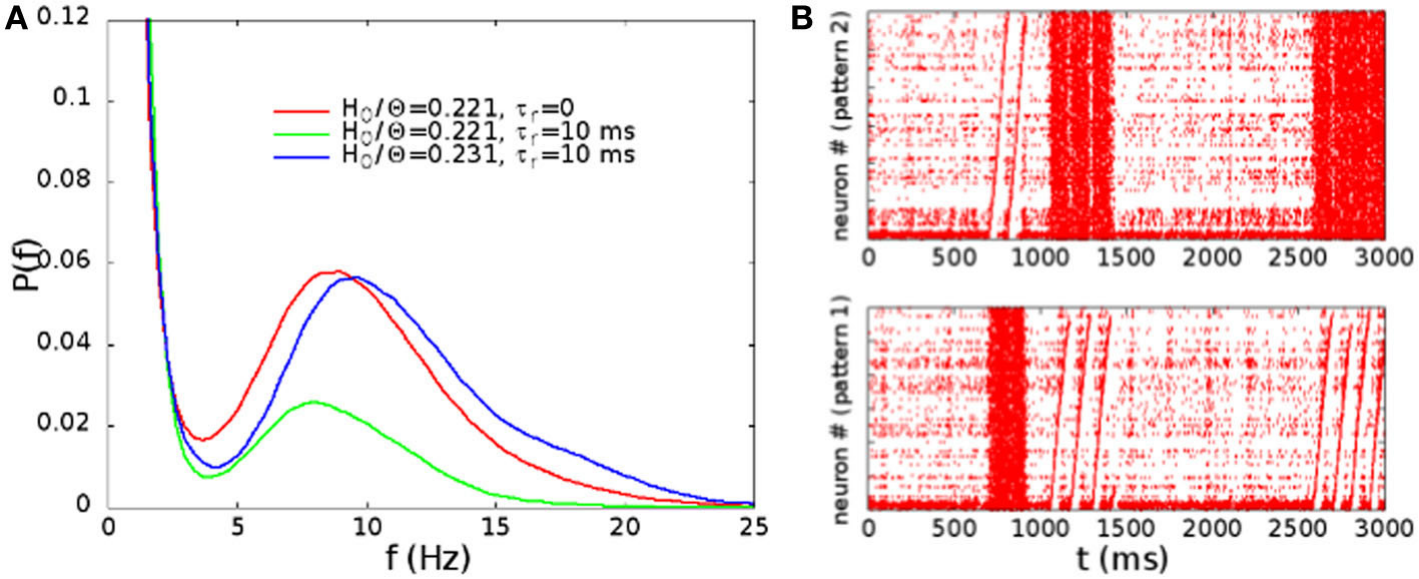
**(A)** Distribution of the rates, evaluated as in Figure 3, in absence of STSD for *H*_0_/Θ = 0.221 (point B in Figure 2), and in presence of STSD for *H*_0_/Θ = 0.221 and 0.231. **(B)** Raster plot of the network with STSD, in the case of *H*_0_/Θ = 0.231 and τ_*r*_ = 10 ms. Alternation of up and down states is similar to the case of Figure 4B.

In Figure 9A, we show the distribution of the rates, evaluated as in Figure 3, in absence of STSD for *H*_0_/Θ = 0.221 (point B in Figure 2), and in presence of STSD for *H*_0_/Θ = 0.221 and 0.231. Note that, for the same synaptic strength at rest, the effect of STSD is to lower the fraction of time in which the network is in the up state. However, for a slightly higher *H*_0_/Θ, the distribution is very similar to the one without STSD. In Figure 9B, we show the raster plot in the case of *H*_0_/Θ = 0.231 and τ_*r*_ = 10 ms, showing a behavior very similar to the one displayed in Figure 4B, with the alternation of up and down states, and of different patterns replayed in the up states. Therefore, the effect of STSD is to slightly shift the region of the phase space in which the transition is observed.

## 4. Discussion

Our model is the first, to our knowledge, that describe both neural avalanches, recurrences of spatiotemporal patterns, and alternation of up and down states, in a single minimal model.

Differently from our previous work (Scarpetta and de Candia, 2013) here we study a sparse connectivity, which is a results of a competitive pruning process applied after the learning procedure. Moreover while we previously introduced heterogeneity in the network topology using neurons with different spiking thresholds, here we show that avalanches initiation may be initiated by the interplay between miniatures noise and the heterogeneity in the strengths of connections, in agreement with recent experimental results (Orlandi et al., 2013).

The model shows a region of low activity, with Poissonian spiking rate, and a region of high activity, characterized by the continuous replay of one of the multiple attractors stored in the network connections, depending on the value of synaptic strength and noise intensity. In the region of phase space separating these two regimes, one observes an alternation of periods of quiescence (down states) and periods of high correlated activity (up states), corresponding to an intermittent replay of the patterns. At a finer temporal scale, up states are made of a sequence of avalanches, showing power law distribution of sizes and durations. In this model the alternation of up and down states does not depend on a kind on neuron bistability, nor on synaptic depression, but is rather a network effect, the result of a structured connectivity, that produces multiple dynamical attractors, and of the fact that at the non-equilibrium phase transition the network dynamics fluctuates between different metastable basins of attraction.

Therefore, such complex dynamics appears at a dynamical transition between disordered Poissonian activity, and an ordered permanent dynamical state. In such region, the network is able to respond to external inputs in a flexible way, switching effectively between different modes of operation, corresponding to the different basins of attraction, that may be connected to functionally relevant behavior.

### Conflict of interest statement

The authors declare that the research was conducted in the absence of any commercial or financial relationships that could be construed as a potential conflict of interest.

## Footnotes

^1^This is the time appearing in Equation (1), not the CPU time needed to perform the simulations, that ranged between 1 and 5 h.

^2^Note that, once the replay of the pattern has started, it is able to sustain itself, provided the synaptic strengths are larger than some threshold (Scarpetta et al., 2010; Scarpetta and Giacco, 2012), so that with a suitable triggering it is possible to observe the continuous replay of the pattern down to α = 0.
